# Comparison of Polytetrafluoroethylene Flat-Sheet Membranes with Different Pore Sizes in Application to Submerged Membrane Bioreactor

**DOI:** 10.3390/membranes2020228

**Published:** 2012-06-01

**Authors:** Tadashi Nittami, Tetsuo Hitomi, Kanji Matsumoto, Kazuho Nakamura, Takaharu Ikeda, Yoshihiro Setoguchi, Manabu Motoori

**Affiliations:** 1 Department of Chemical Engineering, Yokohama National University, 79-5 Tokiwadai, Hodogaya-ku, Yokohama 240-8501, Japan; Email: d08ga638@ynu.ac.jp (T.H.); k-mtmt@ynu.ac.jp (K.M.); naka1@ynu.ac.jp (K.N.); 2 Corporate Research Division, Nippon Valqua Industries, Ltd., 2-2-2 Oyamagaoka, Machida, Tokyo 194-0215 Japan; t-ikeda@valqua.co.jp (T.I.); y-setoguchi@valqua.co.jp (Y.S.); m-motoori@valqua.co.jp (M.M.)

**Keywords:** activated sludge, flat-sheet membrane module, membrane bioreactors, polytetrafluoroethylene (PTFE), wastewater treatment.

## Abstract

This study focused on phase separation of activated sludge mixed liquor by flat-sheet membranes of polytetrafluoroethylene (PTFE). A 20 liter working volume lab-scale MBR incorporating immersed PTFE flat-sheet membrane modules with different pore sizes (0.3, 0.5 and 1.0 μm) was operated for 19 days treating a synthetic wastewater. The experiment was interrupted twice at days 5 and 13 when the modules were removed and cleaned physically and chemically in sequence. The pure water permeate flux of each membrane module was measured before and after each cleaning step to calculate membrane resistances. Results showed that fouling of membrane modules with 0.3 μm pore size was more rapid than other membrane modules with different pore sizes (0.5 and 1.0 μm). On the other hand, it was not clear whether fouling of the 0.5 μm membrane module was more severe than that of the 1.0 μm membrane module. This was partly because of the membrane condition after chemical cleaning, which seemed to determine the fouling of those modules over the next period. When irreversible resistance (*R_i_*) *i.e.*, differences in membrane resistance before use and after chemical cleaning was high, the transmembrane pressure increased quickly during the next period irrespective of membrane pore size.

## 1. Introduction

Membrane bioreactors (MBRs) have been used increasingly in wastewater treatment to minimize solid phase–liquid phase separation problems often encountered in conventional activated sludge clarifiers [1]. MBR systems also have the advantage of operating at high mixed liquid suspended solids (MLSS) concentrations, generating a lower excess sludge production, and the treated water can be reused [2]. In addition, biological nutrient removal (BNR) processes with MBR can be attractive because the plant footprint is reduced from the absence of settling tanks [1]. However, membrane fouling is a major problem in MBR systems. It affects negatively the permeability of the membrane and results in increasing operation and maintenance costs, which has become one of the most important factors hindering the widespread application of MBRs [3,4]. A severely fouled membrane must be cleaned with chemical reagents [5]. Therefore, membranes for MBR application should possess a low fouling-propensity to maintain a high permeate flux, and a high durability from exposure to the chemicals used for cleaning them. 

Membrane fouling is affected by interactions between the membrane and mixed liquor; hence membrane characteristics, such as membrane material, pore size and hydrophobicity, are important factors [6]. Previous experiments using polymeric membranes have revealed a close relationship between membrane material type and fouling [5]. However, little information is available on the impact of polymeric membrane materials on membrane fouling in MBRs [6,7]. In particular, although polytetrafluoroethylene (PTFE) is being used increasingly for membrane construction in MBRs [6,8,9], few studies have focused on their performance characteristics. 

PTFE has several attractive features including a higher chemical resistance, heat resistance and porosity, compared to other membrane materials like polyethylene and polyvinylidene fluoride. Thus, the properties of flat-sheet microfiltration membranes made from PTFE were examined in this study. Symmetrical hydrophobic PTFE flat-sheet membrane modules were used in a lab-scale submerged MBR to understand how their pore size might influence filtration performance.

## 2. Experimental Section

### 2.1. Membrane Characteristics and Experimental MBR Set-Up

**Figure 1:**
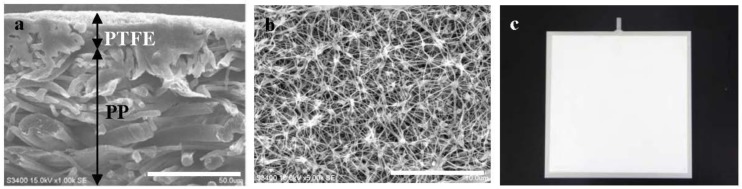
SEM images of PTFE membrane (pore size 0.5 μm) laminated with polypropylene (PP) and its flat-sheet membrane module photo. (**a**) cross-section image with filtration direction from top to bottom (1000×); (**b**) surface image (5000×); (**c**) membrane module. Scale bars in Figure 1**a**, **b** shows 50 μm and 10 μm respectively.

Characteristics of symmetrical hydrophobic PTFE flat-sheet membrane with three different pore sizes (0.3, 0.5 and 1.0 μm) used here are listed in Table 1. The PTFE flat-sheet membranes were laminated with polypropylene (PP) and then attached to both sides of the membrane modules (Figure 1). Total membrane surface area of the membrane module was 0.029 m^2^ (0.12 m × 0.12 m × 2 sides). A lab-scale MBR (*L* × *W* × *H* = 0.4 m × 0.1 m × 0.5 m) was configured with a 20 liter working volume of a reactor tank incorporating four immersed PTFE flat-sheet membrane modules. The filtrate was recovered with a roller pump with a suction mode of 9-min-on and 1-min-off and was returned into the reaction tank. Constant filtrate flux (1.4 ± 0.2 m d^−1^) was maintained by adjustment of the pump rotation rate. The transmembrane pressure (TMP) generated by filtration was measured periodically with pressure gages (AP-51A, Keyence, Osaka, Japan) to investigate the influence of membrane pore size on membrane fouling. The air for washing the membrane surface was supplied continuously at 2 L min^−1^ from a diffuser located directly below the membrane module.

**Table 1:** Characteristics of the polytetrafluoroethylene (PTFE) membranes in this study.

Nominal pore size [μm]	Pore size range [μm] ^a^	Contact angle [°]	Membrane thickness [μm]	Pore morphology	Surface porosity [%]
0.3	0.2–0.4	135	25 ± 10	symmetric	85
0.5	0.4–0.7	135	15 ± 10	symmetric	88
1.0	0.7–1.3	135	10 ± 10	symmetric	90

^a^ Pore sizes were determined by the bubble point method according to ASTM F316-86 (1986) [10].

### 2.2. Synthetic Wastewater and Operational Conditions

The MBR was operated for 19 days incorporating immersed three new PTFE flat-sheet membrane modules with pore sizes 0.3 μm, 0.5 μm and 1.0 μm (PTFE 0.3 μm, 0.5 μm and 1.0 μm (A)) and a used PTFE membrane module with pore size 1.0 μm (PTFE 1.0 μm (B)) under room temperature (15–19 °C). The pH was kept at 6.5–7.5 without control. A concentrated synthetic wastewater (SWW) was prepared, which contained the following (g L^−1^): Glucose, 8.57; CH_3_COONa, 2.96; Bacto Peptone, 4.28; and KH_2_PO_4_, 0.396. The concentrated SWW was made up with milliQ water and sterilized by autoclaving. The SWW was dosed at a constant flow of 0.286 L/day into the reactor tank with a micro tube pump MP-3 (Eyela, Tokyo, Japan). Inoculum was obtained from a 40 L lab-scale batch reactor, which was fed with the same SWW as above for over 10 years. The MBR tank volume was set at 18 L at the beginning of operation and was then increased up to 20 L by adding 2 L of SWW a week. In this study, all the filtrate was returned to the reactor tank, but 2 L of supernatant was withdrawn once a week. Thus, the HRT was controlled at 63–70 days. SRT was uncontrolled with no wasting of the biomass, and so the mixed liquor suspended solids (MLSS) level increased from 6500 to 8500 mg L^−1^. The operation was interrupted twice at days 5 and 13 when the modules were removed from the MBR and washed. Thus, the operational period was divided into three runs: Run 1 (day 0–5), Run 2 (day 5–13), and Run 3 (day 13–19). Note that the membrane module PTFE 1.0 μm (B) was used previously in the same MBR for eight days under the same operational conditions as above, except at a different filtrate flux (0.7 ± 0.1 m d^−1^). It was washed physically and chemically after using as detailed below before being used in this study.

### 2.3. Membrane Cleaning and Membrane Resistance Measurement

The membrane modules were washed sequentially as follows: physical cleaning by wiping surfaces with a soft sponge and chemical cleaning by immersing modules in 2000 mg L^−1^ sodium hypochlorite solution for >5 h. The pure water permeate flux of each membrane module was measured before use, after each operation, and after each physical and chemical cleaning step, to calculate the hydraulic resistance: *R* (m^−1^) by Equation (1). The differences in hydraulic resistance after operation and after physical cleaning, after physical cleaning and after chemical cleaning, and before use and after chemical cleaning, were defined as *R_rp_*, *R_rc_*, and *R_i_*, respectively. The TMP (Pa) was measured with pressure gauges (AP-51A, Keyence, Osaka, Japan). The permeate flow: *J* (m^3^ m^2^ h^−1^) was measured periodically by collecting permeate into a measuring cylinder. The permeate viscosity: *μ* (Pa s) was calculated by assigning the temperature of the activated sludge mixture: *T* (K) to Equation (2) [11].


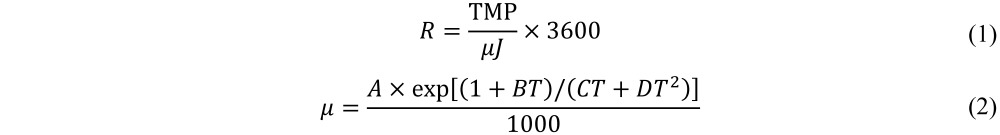

where A, B, C and D are 0.12571873 × 10^−1^, −0.58064362 × 10^−2^, 0.11309108 × 10^−2^, and −0.57239520 × 10^−5^, respectively.

### 2.4. Scanning Electron Microscope (SEM) Analysis

The surfaces of new PTFE membranes (pore sizes 0.3, 0.5 and 1.0 μm) were examined by SEM (S-3400N, Hitachi High-Technologies, Japan) and their membrane modules were immersed in the MBR under the same operational conditions as described above except for the filtrate flux (0.7 ± 0.1 m d^−1^). Modules were washed physically and chemically when the TMP reached 20 kPa, removed from the MBR after 30 days operation and then washed physically as above. These membrane surfaces were also examined by SEM. All membrane samples were dried at 60 °C for 12 h and then sputter-coated with Pt/Pd (80:20) before SEM observation.

### 2.5. Analytical Methods

The mixed liquor suspended solid (MLSS) of activated sludge mixture was measured according to Standard Methods (1998) [12].

## 3. Results and Discussion

### 3.1. Filtration Performance of Each Membrane Module

**Figure 2:**
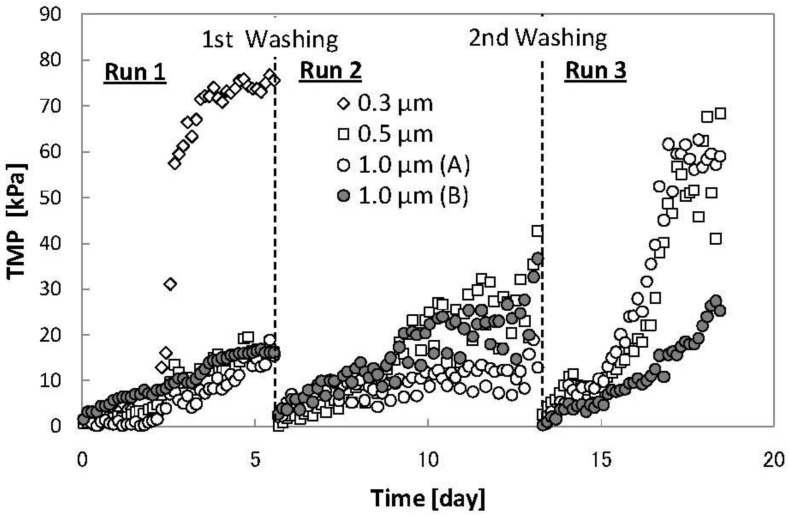
Time course of transmembrane pressure (TMP) of each PTFE membrane module.

Figure 2 shows the changes in TMP of four membrane modules over 19 days. The increase in TMP of membrane module PTFE 0.3 μm was more rapid than seen with the other membrane modules (PTFE 0.5 μm and 1.0 μm (A, B)) during the first five days (Run 1). Therefore, the membrane module PTFE 0.3 μm was removed and the MBR was operated subsequently without it. The increase of TMP of membrane module PTFE 1.0 μm (A) was slower than seen with the membrane modules PTFE 0.5 μm and 1.0 μm (B) during Run 2, whereas membrane module PTFE 1.0 μm (B) fouled slower than others during Run 3. Moreover, the increase of TMP of the membrane module PTFE 0.5 μm was similar to that of the membrane module PTFE 1.0 μm (B) and (A) during Runs 2 and 3. These results suggest that the 0.3 μm membrane had a remarkably high fouling propensity compared to the others, although it was not clear whether fouling of the 0.5 μm membrane was more severe than that of the 1.0 μm membranes or whether the used 1.0 μm membrane (B) fouled more severely than the new 1.0 μm membrane (A).

Van der Marel *et al*. [13] investigated the influence of membrane pore size on membrane fouling using polymeric flat-sheet membranes with different properties from a pilot-scale MBR. They reported that for reasons not explained, symmetric mixed cellulose ester (MCE) membranes with 0.8 μm pore size had excellent performance criteria compared to membranes with 0.1, 1.8 and 2.7 μm pore sizes. The MCE 0.8 μm membrane showed a much lower increase in initial membrane resistance with an increase in flux of 5 to 100 L m^−2^ h^−1^ over 135 h operation. As mentioned above, the pore morphology of the PTFE membranes used in this study was also symmetric. Membranes with 0.5 and 1.0 μm pore sizes showed a similar performance to each other and an improved performance to the 0.3 μm pore size membranes. This outcome would suggest that the optimum pore size for symmetrical membranes is 0.5–1.0 μm.

### 3.2. Membrane Resistance of Each Module

**Figure 3:**
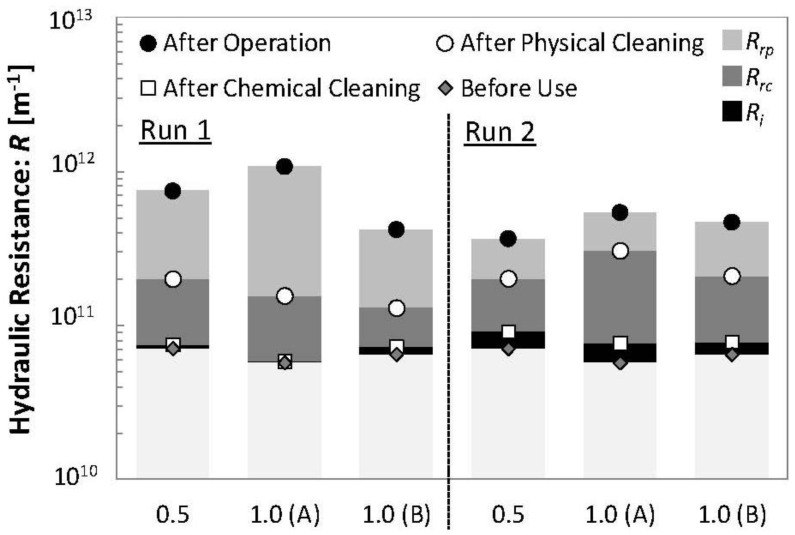
Hydraulic resistance: *R* (m^−1^) and its differences in each step at Run 1 and 2 in each PTFE membrane module. *R_rp_*, *R_rc_* and *R_i_* mean the differences in membrane resistance after operation and after physical cleaning, after physical cleaning and after chemical cleaning, and before use and after chemical cleaning respectively.

The pure water permeate fluxes of each PTFE membrane module (0.5 and 1.0 μm pore size) at each stage were measured and the hydraulic resistance: *R* (m^−1^) calculated (Figure 3). The *R* values are shown as a scatter chart and the differences in *R* values as bar graphs in Figure 3. The *R* values of the three modules showed different patterns during Run 1. This suggests that no correlation exists between *R* values or their differences and increases in TMP during earlier membrane cleaning periods, since TMP increases were very similar for all three pore sizes during Run 1 (Figure 2). However, the *R* values differences and increases in TMP at the next period of membrane cleaning seemed to correlate. The irreversible resistances, *R_i_*, i.e., differences in membrane resistance before use and after chemical cleaning, in membrane modules PTFE 1.0 μm (A) and (B) were clearly lower than those in other modules after Run 1 and Run 2 respectively. As mentioned above, increases in the TMP of membrane modules PTFE 1.0 μm (A) and (B) were slower than those seen with other modules during Run 2 and Run 3 respectively. When *R_i_* was high, TMP seemed to increase rapidly during the next operational period irrespective of membrane pore size. Figure 3 also shows that differences in membrane resistances after operation and after physical cleaning (reversible resistance by physical cleaning: *R_rp_*) seemed to correlate with *R_i_ i.e.*, the higher the *R_rp_*, the lower the *R_i_*. The *R_rp_* in membrane modules PTFE 1.0 μm (A) and (B) were higher than those seen in the other modules during Run 1 and Run 2.

### 3.3. Correlation between Membrane Resistances and Transmembrane Pressure

**Figure 4:**
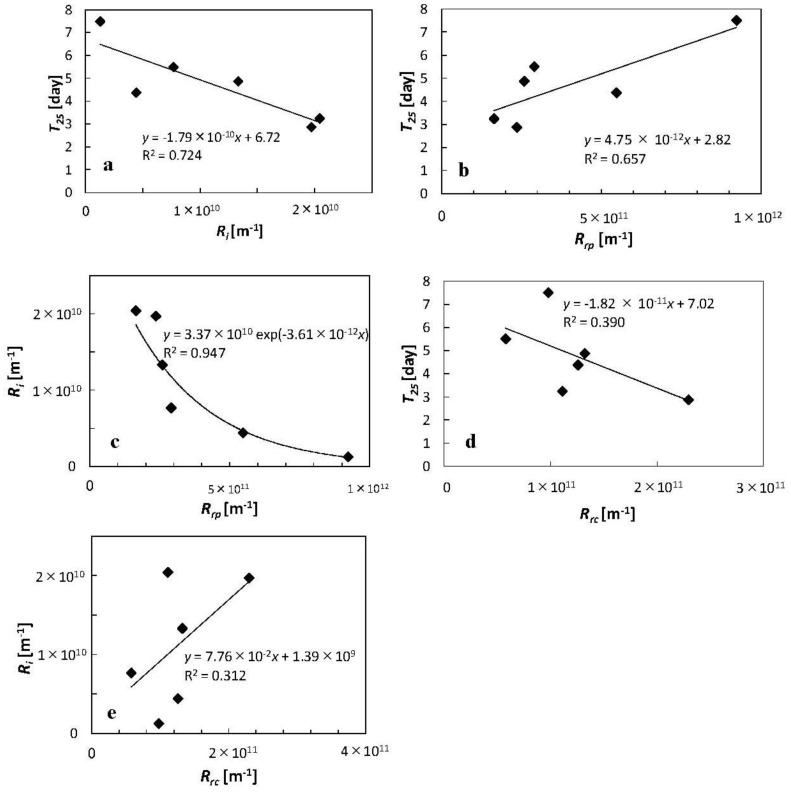
Correlation between the TMP, *R_i_* and *R_rp_*. (**a**) correlation between dates required for increasing TMP up to 25 kPa (*T*_25_) and *R_i_*; (**b**) correlation between *T*_25_ and *R_rp_*; (**c**) correlation between *R_i_* and *R_rp_*; (**d**) correlation between *T*_25_ and *R_rc_*; (**e**) correlation between *R_i_* and *R_rc_*.

To discuss the correlation between TMP increases and *R_i_* and *R_rp_*, data from Runs 2 and 3 (Figure 2) and Runs 1 and 2 (Figure 3) were rearranged in Figure 4a–e. Figure 4a shows the correlation between dates required to increase TMP to 25 kPa (*T_25_*) and *R_i_*. The determination coefficient (*R*^2^) of collinear approximation was low (0.724), although the decrease in *T_25_* seen with an increase of *R_i_* was very apparent. This suggests that *R_i_* of the module may determine its permeate ability during its next operation irrespective of membrane pore size although *T_25_* values may depend not only on *R_i_* of the module but also on MBR operational conditions such as foulants concentration in the MBR system. Figure 4b shows the correlation between *T_25_* and *R_rp_*. The *R*^2^ of collinear approximation was even lower (0.657), although again the increase in *T_25_* with an increase of *R_rp_* was clearly evident. Figure 4c also shows the relationship between *R_i_* and *R_rp_*. The *R*^2^ of exponential approximation (0.947) was higher than that of collinear approximations in Figure 4a,b, and the decrease of *R_i_* with increases in *R_rp_* was clear. These results suggest that the cake or gel layer formed on the surface of membrane and which was removed by physical cleaning was preventing foulants access responsible for high *R_i_* from contaminating the membrane pores. This cake or gel layer may play a key role as also reported in other dynamic MBR studies [14]. On the other hand, a low correlation was seen between *T_25_* and reversible resistance by chemical cleaning (*R_rc_*) or *R_i_* and *R_rc_* (Figure 4d,e). The *R^2^* of collinear approximation between *T_25_* and *R_rc_*, and *R_i_* and *R_rc_* were 0.390 and 0.312 respectively.

### 3.4. SEM Examination of Membrane Surfaces

**Figure 5:**
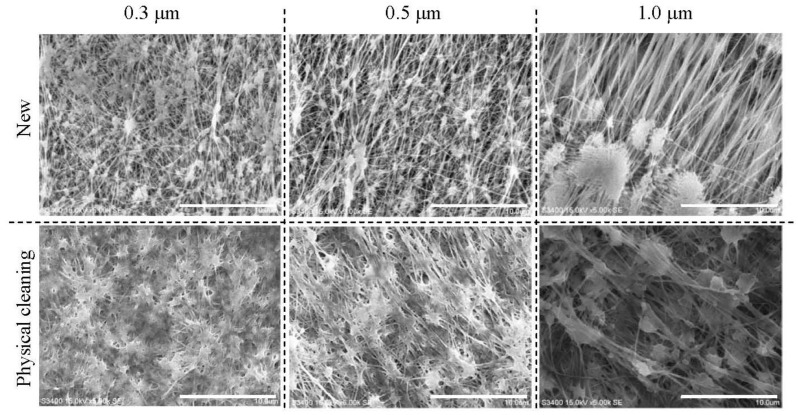
PTFE membranes with pore size of 0.3, 0.5 and 1.0 μm (5000×) before use (new membrane) and after physical cleaning. Scale bars show 10.0 μm respectively.

Figure 5 shows SEM images of membrane surfaces before use (new membrane) and after physical cleaning, and demonstrates that the 0.3 μm pore sized membrane after physical cleaning was more severely fouled internally than the 0.5 and 1.0 μm pore sized membranes, consistent with the rapid TMP increase of membrane module PTFE 0.3 μm measured during Run 1 (Figure 2).

## 4. Conclusions

The results here show that the fouling of module with 0.3 μm pore membrane was more rapid than that seen with other membrane modules with different pore sizes (0.5 and 1.0 μm), and confirmed by SEM observations. On the other hand, condition of the membrane after chemical cleaning seemed to determine their subsequent fouling. Thus, if the *R_i_*, *i.e.*, differences in membrane resistance before use and after chemical cleaning was high, the TMP then increased quickly, irrespective of membrane pore size (0.5 and 1.0 μm). Furthermore, the *R_rp_* correlated with *R_i_* so that the higher the *R_rp_*, the lower the *R_i_*. This suggests that the cake or gel layer formed on the surface of membrane protects the membranes from further fouling.
